# A Cross-Sectional Study of the Relationship Between Exercise, Physical Activity, and Health-Related Quality of Life Among Japanese Workers

**DOI:** 10.3389/fspor.2022.809465

**Published:** 2022-02-24

**Authors:** Ryosuke Sugano, Kazunori Ikegami, Hisashi Eguchi, Mayumi Tsuji, Seiichiro Tateishi, Tomohisa Nagata, Shinya Matsuda, Yoshihisa Fujino, Akira Ogami

**Affiliations:** ^1^Department of Work Systems and Health, Institute of Industrial Ecological Sciences, University of Occupational and Environmental Health, Kitakyushu, Japan; ^2^Department of Mental Health, Institute of Industrial Ecological Sciences, University of Occupational and Environmental Health, Kitakyushu, Japan; ^3^Department of Environmental Health, School of Medicine, University of Occupational and Environmental Health, Kitakyushu, Japan; ^4^Department of Occupational Medicine, School of Medicine, University of Occupational and Environmental Health, Kitakyushu, Japan; ^5^Department of Occupational Health Practice and Management, Institute of Industrial Ecological Sciences, University of Occupational and Environmental Health, Kitakyushu, Japan; ^6^Department of Public Health, School of Medicine, University of Occupational and Environmental Health, Kitakyushu, Japan; ^7^Department of Environmental Epidemiology, Institute of Industrial Ecological Sciences, University of Occupational and Environmental Health, Kitakyushu, Japan

**Keywords:** exercise, physical activity, health-related quality of life, self-rated health, worker

## Abstract

**Background:**

Studies have determined that exercise and physical activity positively affect physical and mental health, and that healthy workers contribute to increased work performance. The relationship between the time spent on exercise during leisure time and physical activity, including work, with health-related quality of life (HRQOL) in workers is unclear, with variations observed between occupational types. This cross-sectional study examined these associations among Japanese workers from various occupations during the COVID-19 pandemic.

**Methods:**

An Internet-based national health survey—Collaborative Online Research on Novel-coronavirus and Work-study (CORoNaWork study)—was conducted among 33,087 Japanese workers in December 2020. After excluding invalid responses, 27,036 participants were categorized into four and five groups according to exercise and physical activity time, respectively. Each group's scores were compared on each of the four questions on the Japanese version of the Centers for Disease Control and Prevention Health-Related Quality of Life (CDC HRQOL-4) using generalized linear models. Age-sex adjusted and multivariate models were used to compare each index of the CDC HRQOL-4.

**Results:**

Compared to the reference category (almost never), any level of exercise (ORs 0.56–0.77) and physical activity (ORs 0.93–0.88) were associated with better self-rated health in the multivariate model. Any exercise was also associated with significantly reduced odds for physically or mentally unhealthy days; however, high levels of physical activity (≥120 min/day) were associated with significantly increased odds for these outcomes (ORs = 1.11 and 1.16, respectively).

**Conclusions:**

The results suggest that exercise habits are more critical to workers' HRQOL than physical activity. Interventions that encourage daily exercise even for a short time are likely to be associated with better workers' health and work performance.

## Introduction

People who frequently engage in physical activity and have good exercise habits have good physical and mental health (Goodwin, 2003; Inoue et al., 2008; Schuch et al., 2016; Bull et al., 2020). Furthermore, studies regarding the occupational field show that absenteeism due to health problems decreased with increased physical activity (López-Bueno et al., 2020); additionally, regular aerobic exercise and strength training prevented work productivity loss due to presenteeism (Walker et al., 2017a), increased physical activity and reduced work restrictions (Walker et al., 2017b). Therefore, we believe that workers require appropriate exercise habits for good health and enhanced work performance.

The coronavirus disease 2019 (COVID-19) pandemic significantly and globally impacted daily life and work in 2021. In Japan, the COVID-19 infection spread rapidly in April 2020. Initially declaring a state of emergency in several prefectures on April 7, 2020, the government expanded it to the whole country on April 16, 2020. Under this declaration, people were urged to avoid the three Cs (closed space with poor ventilation, crowded places with many people nearby, and close-contact settings, such as close-range conversations) and refrain from going out to reduce contact opportunities by 80%. Additionally, to prevent the spread of infection, facilities where many people gather, such as department stores, movie theaters, and sports clubs and playgrounds were requested to be closed (Office for Novel Coronavirus Disease Control CS Government of Japan, 2021).

A study conducted in China in February 2020 reported that about 75% of the target population participated in physical activity at least twice a week before the pandemic; however, 65% of the population had low physical activity levels during the pandemic (Qi et al., 2020). Additionally, in a survey conducted among 5,000 Japanese in February 2020, 40.3% of respondents reported decreased physical activity compared to the same month the previous year (before the pandemic) (Yamada et al., 2020). Another study showed that the COVID-19 home confinement negatively affected physical activity (Ammar et al., 2020). Considering the above, it can be discerned that the state of emergency implemented in April 2020 affected people's exercise habits and reduced physical activities, such as walking, in Japan.

As recognized, exercise and physical activity are associated not only with diseases and work performance but also with the quality of life (QOL). For example, studies have shown that increased physical activity, higher energy physical activity, and exercise interventions are associated with a higher quality of life in older adults, pregnant women, young adult cancer patients, and colorectal cancer patients (Phillips et al., 2013; Krzepota et al., 2018; Zhi et al., 2019; Singh et al., 2020). As for workers, a meta-analysis of physical activity and HRQOL among office workers found that physical activity improved the latter (Nguyen et al., 2021). One study reported a positively correlation between the frequency of physical activity among construction workers and HRQOL (Malak, 2017). Conversely, a study investigating the relationship between lifestyle and QOL of manufacturing industry workers showed that the low QOL group had significantly longer physical activity time than the high QOL group (Rabanipour et al., 2019). Therefore, we believe that further research on the relationship between exercise/physical activity and QOL of workers, regardless of their occupation, is needed. In the present study, we focused on the relationship between exercise, physical activity, and health-related quality of life (HRQOL), which is defined as an individual's or group's perceived physical and mental health over time (Centers for Disease Control Prevention of USA, 2021a).

Understanding the relationship between the time spent exercising during leisure, time spent engaged in physical activity, including work, and HRQOL will provide important information for developing interventions to improve workers' health and work performance. Furthermore, we thought that it would be useful to investigate these relationships during the COVID-19 pandemic, mainly because physical activity tends to be restricted. As such, this study investigated the relationship between exercise, physical activity, and HRQOL using a large-scale Internet survey of workers conducted during the COVID-19 pandemic.

## Methods

### Study Design and Setting

The present cross-sectional study used a part of the baseline data of a prospective cohort study conducted by a research group at the University of Occupational and Environmental Health in Japan. The study is called the Collaborative Online Research on Novel-coronavirus and Work-study (CORoNaWork study). A Japanese Internet survey company—Cross Marketing Inc. Tokyo—implemented the study, which involved a self-administered questionnaire survey. Baseline survey data were collected from December 22–25, 2020. Incidentally, the baseline survey occurred during the third wave of the pandemic when the number of COVID-19 infections and deaths was overwhelmingly higher than in the first and second waves; therefore, Japan was on high alert. The details of the study protocol are provided by Fujino et al. (2021).

### Participants

The participants were between 20 and 65 years and working at the time of the baseline survey. A total of 33,087 adults participated in the CORoNaWork study, which employed stratified cluster sampling by gender, age, region, and occupation. A database of 27,036 individuals was created by excluding 6,051 who were determined to have provided invalid responses. Invalid responses include abnormally short response time (<6 min), abnormally short height (<140 cm), abnormally low weight (<30 kg), different answers to similar questions in the survey (e.g., inconsistent answers to questions about living with family members), and incorrect answers to questions used to identify inappropriate responses (e.g., choosing the third-highest number from the following five numbers).

### Questionnaire

The questionnaire items are described in detail by Fujino et al. (2021). We used questionnaire data on sex, age, presence of illness under medical treatment, average exercise time during leisure time per day, average physical activity time including work per day, educational background, area of participants' residence, job type, working hours per day, telecommuting frequency, and HRQOL. Exercise time during leisure time per day, and average physical activity time, including work per day, were ascertained based on the following question: “On average, how much time do you spend on the following activities?—Exercise time/Physical activity (including work).” The response options included more than 2 h/more than 1 h/more than 30 min/ <30 min/almost never. These questions were asked in a matrix format to make it easier for the participants to answer. We randomly assigned half of the subjects to each group to check the reliability of the responses between subjects and found no significant difference in the means [SD] between the two groups for either the exercise or physical activity questions (3.11 [1.1] vs. 3.11 [1.1], *p* = 0.984 and 3.15 [1.6] vs. 3.16 [1.6], *p* = 0.724, respectively). Moreover, a validity study using the results of a 24-h physical record and questions about activity during work and leisure times showed a moderate to strong correlation between the two values (Fujii et al., 2011). From this result, we believe that the questions in this study have a certain degree of validity.

The CDC HRQOL-4, developed by the Centers for Disease Control and Prevention (CDC) as a tool for public health surveillance (Centers for Disease Control, 2001), was used to assess overall HRQOL. The CDC HRQOL-4 includes the following four items: (a) self-rated health, (b) the number of physically unhealthy days in the past 30 days, (c) the number of mentally unhealthy days in the past 30 days, and (d) the number of days with activity limitation in the past 30 days. Self-rated health was recorded as excellent, very good, good, fair, or poor and ranged from a minimum score of 1 to a maximum of 5, with lower scores indicating better self-rated health (Centers for Disease Control Prevention of USA, 2021b).

International studies have provided support for the reliability and validity of the CDC HRQOL-4. In 2020, the Japanese version of the CDC HRQOL-4 was developed and evaluated for its reliability and validity for use with workers (Chimed-Ochir et al., 2020a). Furthermore, because the CDC HRQOL-4 was found to be associated with indicators of work functioning impairment (Chimed-Ochir et al., 2020b), the measurement of HRQOL is helpful for both health and labor-management assessment of workers.

### Outcomes and Measures

As for outcome variables, we used CDC HRQOL-4 scores for self-rated health (5-point Likert scale: 1 = excellent, 2 = very good, 3 = good, 4 = fair, and 5 = poor) and the number of days in the past 30 days reported for physically unhealthy days, mentally unhealthy days, and activity limitation days. The number of physically unhealthy days, mentally unhealthy days, and activity limitation days were categorized as more than 5 days and <5 days based on the 75th percentile value of physically unhealthy days and mentally unhealthy days.

As for the exposure variables, we used exercise time during leisure and physical activity time, including work. Based on the responses to the questionnaire, the participants were divided into groups. For exercise time during leisure, there were only a few responses of more than 2 h; accordingly, we classified them into four groups: ≥60 min/day, 30–59 min/day, 1–29 min/day, and almost never. Participants were also classified into five groups according to their physical activity time, including work: ≥120 min/day, 60–119 min/day, 30–59 min/day, 1–29 min/day, and almost never. The following items were used as confounding factors. Sex, age (20–29, 30–39, 40–49, 50–59, and ≥60 years of age), educational background (junior or senior high school, junior college, or vocational school, university, or graduate school), and presence of illness under medical treatment were used as personal characteristics. Job type (desk work, hospitality work, manual work), telecommuting frequency (4 days/week or more, 2–3 days/week, 1 day/week or less, almost never), working hours per day (<8 h/day, 8–9 h/day, 9–11 h/day, 11 h/day or more) were used as work-related factors.

### Statistical Method

To analyze the four domains of CDC HRQOL-4 among the four groups of exercise time or the five groups of physical activity time, we used generalized linear models (GLMs). They included logistic regression and ordinal logistic regression, with two models of the sex-age-adjusted model and the multivariate model. For the multivariate model, we added variables related to educational background, presence of illness under medical treatment, job type, telecommuting frequency, and working hours per day as explanatory variables to GLMs. In all the tests, the threshold for significance was set at *P* < 0.05. We used SPSS 25.0 J analytical software (IBM, NY, USA) for statistical analyses.

## Results

### Participants and Descriptive Data

A total of 13,814 (51.1%) respondents were men, and the mean age was 47.0 years (SD: 10.5). Of the total population, 13,171 (48.7%) workers had a university or graduate school education, 13,468 (49.8%) were engaged in desk work, and 21,276 (78.7%) did not telecommute. As for exercise during leisure, 3,202 (11.8%) participants spent 60 min/day or more, 4,210 (15.6%) spent 30–59 min/day, 6,123 (22.6%) spent 1–29 min/day, and 13,501 (49.9%) reported they almost never exercised. As for physical activity time including work, 6,843 (25.3%) participants spent more than 120 min/day, 3,062 (11.3%) spent 60–119 min/day, 4,257 (15.7%) spent 30–59 min/day, 4,770 (17.6%) spent 1–29 min/day, and 8,104 (30.0%) reported they almost never spent time engaged in physical activity (Table 1). The relationship between exercise time and physical activity time is presented in Table 2. Participants with almost never physical activity tended to have never exercised. The group spent more than 120 min/day in physical activity time. Notably, the percentage with almost never exercise time was the largest.

**Table 1:** Participants' characteristics.

**Items**	***n*** **(%)/M (SD)**	
*N*	27,036	(100)
Sex, male	13,814	(51.1)
**Age**		
20–29	1,905	(7.0)
30–39	4,858	(18.0)
40–49	8,011	(29.6)
50–59	9,012	(33.3)
≥60	3,250	(12.0)
**Education**		
Junior or senior high school	7,321	(27.1)
Junior college or vocational school	6,544	(24.2)
University or graduate school	13,171	(48.7)
Presence of Illnesses that require hospital treatment.	9,510	(35.2)
**Job type**		
Desk work	13,468	(49.8)
Hospitality work	6,927	(25.6)
Manual work	6,641	(24.6)
**Telecommuting frequency**		
≥4 days/week	2,790	(10.3)
2–3 days/week	1,477	(5.5)
≤ 1 day/week	1,493	(5.5)
None	21,276	(78.7)
**Working hours per day**		
<8 h/day	5,334	(19.7)
8–9 h/day	14,848	(54.9)
9–11 h/day	5,541	(20.5)
≥11 h/day	1,313	(4.9)
**Exercise time in leisure time**		
≥60 min/day	3,202	(11.8)
30–59 min/day	4,210	(15.6)
1–29 min/day	6,123	(22.6)
Almost never	13,501	(49.9)
**Physical activity time including work**		
≥120 min/day	6,843	(25.3)
60–119 min/day	3,062	(11.3)
30–59 min/day	4,257	(15.7)
1–29 min/day	4,770	(17.6)
Almost never	8,104	(30.0)
**CDC HRQOL−4**		
Self-rated health	3.48	(0.93)
Physically unhealthy days, ≥5 days/month	7,395	(27.4)
Mentally unhealthy days ≥5 days/month	7,010	(25.9)
Days with activity limitation ≥5 days/month	3,686	(13.6)

*CDC HRQOL−4: The Centers for Disease Control and Prevention health-related quality of life−4*.

**Table 2:** Prevalence of exercise time in leisure time and physical activity time including work.

		**Total**	**Exercise time in leisure time**								***p***
			**≥60 min/day**		**30–59 min/day**		**1–29 min/day**		**Almost never**		
			***n***	**(%)**	***n***	**(%)**	***n***	**(%)**	***n***	**(%)**	
Physical activity time including work	≥120 min/day	6,843	938	(13.7)	1,027	(15.0)	1,504	(22.0)	3,374	(49.3)	<0.001
	60–119 min/day	3,062	1,047	(34.2)	565	(18.5)	569	(18.6)	881	(28.8)	
	30–59 min/day	4,257	520	(12.2)	1,570	(36.9)	921	(21.6)	1,246	(29.3)	
	1–29 min/day	4,770	329	(6.9)	596	(12.5)	2,127	(44.6)	1,718	(36.0)	
	Almost never	8,104	368	(4.5)	452	(5.6)	1,002	(12.4)	6,282	(77.5)	

### Comparison of the Scores on the CDC HRQOL-4 Among the Exercise Time Groups

In the age–sex-adjusted and multivariate models, we used GLMs to compare the CDC HRQOL-4 scores for each of the four groups according to exercise time (Tables 3, 4). In the multivariate model for self-rated health (lower scores indicating better self-rated health), compared to the almost never group, all groups showed significantly lower odds ratios (OR [95%CI]); ≥60 min/day (0.56 [052–0.60]), 30–59 min/day (0.67 [0.63–0.71]), and 1–29 min/day (0.77 [0.72–0.81]). For ≥5 days of physically and mentally unhealthy days, all groups showed significantly lower odds ratios than the almost never group. For ≥5 days with activity limitation, the 30–59 and 1–29 min/day groups showed significantly lower odds ratios compared to the almost never group (0.87 [0.78–0.97], 0.84 [0.76–0.93]).

**Table 3:** Odds ratios of self-rated health for exercise time in leisure time and physical activity time including work.

**Parameters**	**Items/Options**	***n***	**M**	**(SD)**	**Age-sex adjusted**			Multivariate*		
					**OR**	**95% CI**	***p***	**OR**	**95% CI**	***p***
Self-rated health	**Exercise time in leisure time**									
	60 min/day	3,202	3.25	(1.01)	0.60	[0.51–0.60]	<0.001	0.56	[0.52–0.60]	<0.001
	30–59 min/day	4,210	3.35	(0.96)	0.71	[0.62–0.71]	<0.001	0.67	[0.63–0.71]	<0.001
	1–29 min/day	6,123	3.44	(0.91)	0.81	[0.72–0.81]	<0.001	0.77	[0.72–0.81]	<0.001
	Almost never	13,501	3.59	(0.90)	Reference			Reference		
	**Physical activity time including work**									
	120 min/day	6,843	3.51	(0.94)	0.99	[0.93–1.05]	0.736	0.93	[0.88–1.00]	0.037
	60–119 min/day	3,062	3.34	(0.97)	0.81	[0.75–0.88]	<0.001	0.8	[0.74–0.87]	<0.001
	30–59 min/day	4,257	3.39	(0.94)	0.85	[0.79–0.91]	<0.001	0.84	[0.78–0.90]	<0.001
	1–29 min/day	4,770	3.45	(0.91)	0.88	[0.82–0.94]	<0.001	0.88	[0.82–0.94]	<0.001
	Almost never	8,140	3.57	(0.91)	Reference			Reference		

*OR, odds ratio; CI, confidence interval*.

* *Multivariate are adjusted for age, sex, education, job type, telecommuting frequency, and working hours per day*.

*Self-rated health takes a range from a minimum of 1 to a maximum of 5, with lower scores indicating better self-rated health*.

**Table 4:** Odds ratios of unhealthy days for exercise time in leisure time, and physical activity time including work.

**Parameters**	**Items/Options**	***n***	**≥5 days**	**Age-sex adjusted**			Multivariate*		
			**%**	**OR**	**95% CI**	***p***	**OR**	**95% CI**	***p***
Physically unhealthy days	**Exercise time in leisure time**								
	60 min/day	3,202	26.0	0.86	[0.79–0.95]	0.002	0.88	[0.80–0.97]	0.008
	30–59 min/day	4,210	25.3	0.83	[0.77–0.91]	<0.001	0.83	[0.77–0.91]	<0.001
	1–29 min/day	6,123	25.9	0.88	[0.82–0.94]	<0.001	0.88	[0.82–0.95]	0.001
	Almost never	13,501	28.9	Reference			Reference		
	**Physical activity time including work**								
	120 min/day	6,843	30.3	1.18	[1.09–1.27]	<0.001	1.11	[1.03–1.20]	0.007
	60–119 min/day	3,062	27.4	1.09	[0.98–1.19]	0.129	1.05	[0.95–1.18]	0.302
	30–59 min/day	4,257	26.4	1.05	[0.96–1.16]	0.23	1.05	[0.96–1.16]	0.270
	1–29 min/day	4,770	25.0	0.99	[0.91–1.08]	0.832	0.99	[0.91–1.09]	0.883
	Almost never	8,140	26.8	Reference			Reference		
Mentally unhealthy days	**Exercise time in leisure time**								
	60 min/day	3,202	23.7	0.81	[0.73–0.88]	<0.001	0.82	[0.74–0.9]	<0.001
	30–59 min/day	4,210	23.6	0.81	[0.75–0.89]	<0.001	0.82	[0.75–0.9]	<0.001
	1–29 min/day	6,123	23.9	0.85	[0.79–0.92]	<0.001	0.85	[0.79–0.93]	<0.001
	Almost never	13,501	28.1	Reference			Reference		
	**Physical activity time including work**								
	120 min/day	6,843	30.1	1.20	[1.12–1.3]	<0.001	1.16	[1.08–1.25]	<0.001
	60–119 min/day	3,062	26.0	1.08	[0.97–1.19]	0.176	1.06	[0.96–1.18]	0.256
	30–59 min/day	4,257	23.8	0.98	[0.89–1.08]	0.701	0.99	[0.89–1.09]	0.768
	1–29 min/day	4,770	22.0	0.91	[0.83–1.00]	0.044	0.92	[0.84–1.01]	0.066
	Almost never	8,140	25.8	Reference			Reference		
Days with activity limitation	**Exercise time in leisure time**								
	60 min/day	3,202	14.1	0.97	[0.86–1.10]	0.646	0.99	[0.88–1.12]	0.889
	30–59 min/day	4,210	12.7	0.86	[0.78–0.96]	0.010	0.87	[0.78–0.97]	0.013
	1–29 min/day	6,123	12.1	0.84	[0.76–0.93]	<0.001	0.84	[0.76–0.93]	0.001
	Almost never	13,501	14.5	Reference			Reference		
	**Physical activity time including work**								
	120 min/day	6,843	14.0	0.99	[0.90–1.09]	0.833	0.93	[0.84–1.03]	0.148
	60–119 min/day	3,062	15.1	1.12	[0.99–1.28]	0.063	1.10	[0.97–1.25]	0.142
	30–59 min/day	4,257	13.5	1.04	[0.93–1.18]	0.478	1.04	[0.93–1.18]	0.462
	1–29 min/day	4,770	12.1	0.96	[0.86–1.08]	0.513	0.97	[0.86–1.1]	0.638
	Almost never	8,140	13.7	Reference			Reference		

*OR, odds ratio; CI, confidence interval*.

* *Multivariate are adjusted for age, sex, education, job type, telecommuting frequency, and working hours per day*.

*>5 days: Percentage of participants with five or more unhealthy days*.

### Comparison of the Scores on the CDC HRQOL-4 Among the Physical Activity Time Groups

The age–sex-adjusted and multivariate models were also used to compare the CDC HRQOL-4 scores for each of the five groups according to physical activity time (Tables 3, 4). In the multivariate model for self-rated health, compared to the almost never group, all groups showed significantly lower odds ratios (OR [95%CI]); ≥120 min/day (0.93 [0.88–1.00]), 60–119 min/day (0.80 [0.74–0.87]), 30–59 min/day group (0.84 [0.78–0.90]), and 1–29 min/day group (0.88 [0.82–0.94]). For ≥5 days of physically and mentally unhealthy days, compared to the almost never group, the ≥120 min/day group showed significantly higher odds ratios (1.11 [1.03–1.20] and 1.16 [1.08–1.25]).

## Discussion

This study clarified the relationship between HRQOL (self-rated health and unhealthy days), exercise time during leisure, and physical activity time, including work. The relationship between exercise time and HRQOL showed that better self-rated health was associated with an increase in time spent exercising, even after adjusting for personal characteristics and work-related factors. Furthermore, the results suggest that exercise, even for a short time, is associated with fewer physically and mentally unhealthy days. The results of this study are similar to those of previous studies conducted on adults in various regions (Brown et al., 2004; Södergren et al., 2008; Abuladze et al., 2017; Hsieh et al., 2018). For instance, a study has shown that those with poor self-rated health had approximately twice the risk of mortality compared to those with good self-rated health (DeSalvo et al., 2006). This implies that even short exercise sessions may contribute to better health and fewer physically and/or mentally unhealthy days. These results provide valuable information while considering interventions to address exercise habits among workers.

Regarding the relationship between physical activity, including work, and HRQOL, more than 120 min/day had a lower impact on self-rated health compared to <120 min/day; moreover, it was associated with an increase in the number of physically and mentally unhealthy days compared to the almost never group. The results are comparable to a study that found no physical activity, more than 90 min of physical activity, and a high frequency of physical activity associated with an increase in unhealthy days (Brown et al., 2004). Regarding physical activity time and mental health, a previous study found that more exercise time during leisure was negatively associated with depression; however, it was not significantly associated with more work-related physical activity time (Munehiro et al., 2016). Other studies have shown that strenuous exercise negatively affects the immune system, such as IL-2 production and leukocyte responses (Shephard et al., 1994) and that increased physical activity increases the risk of musculoskeletal disorders (Hootman et al., 2001). Therefore, physical activity that exceeds 120 min/day may not be appropriate, as it is associated with reduced HRQOL.

For the days of activity limitation, the results suggest an association between exercise time of fewer than 60 min/day and a decrease in the days of activity limitation. In comparison, no such association was found for exercise time of 60 min or more. This relationship is different from those of other domains of HRQOL (self-rated health, physically unhealthy days, and mentally unhealthy days). This difference may be because daily exercise for a long time increases the risk of injury that may interfere with daily life. Studies have shown that the average recreational runner suffers an injury rate of 2.5–12.1/1,000 h during running, which is one of the most popular forms of exercise (van Mechelen, 1992).

These results indicate that HRQOL may be more strongly related to exercise time during leisure than physical activity time, including work. In particular, having an exercise habit, even for a short time, was associated with good HRQOL. As for physical activity time, including work, longer periods may be associated with lower HRQOL. We found that a large percentage of workers who spend much time in physical activity, including work, tend to do little or no exercise. Therefore, encouraging workers to have a short exercise habit, regardless of their level of physical activity, including work, could benefit their health and work performance.

According to a study of Japanese workers, 67% do not inculcate the exercise habits recommended by the Ministry of Health, Labor, and Welfare (Matsuo and So, 2021). Another study has shown that public health restrictions related to COVID-19 have further reduced the amount of time spent outdoors (Cindrich et al., 2021). Additionally, approximately 70% of the participants showed a moderate or greater change in physical activity when voluntary restraint of physical movements was recommended (Balanzá-Martínez et al., 2021). Therefore, even as the COVID-19 pandemic continues, it may be a good idea to recommend that exercises be done indoors. For example, interventions—such as sending fitness equipment, watching videos of exercises on YouTube, text messages from health coaches, exercising with home video game consoles, setting up an ergometer at home, and combining home instruction with unsupervised exercise—lead to increased physical activity, continued exercise, and favorable attitudes toward exercise (Halse et al., 2015; Wherry et al., 2019; Tripicchio et al., 2021). Thus, these practices may lead to the formation of exercise habits, thereby resulting in better HRQOL.

## Limitations

This study has several limitations. First, the generalizability of the results is unclear because the CORoNaWork study is an Internet-based survey. However, to reduce sampling bias, sampling was conducted by generation, residence, and occupation. Second, because this was a cross-sectional study, the causal relationship between exercise time, physical activity time, and HRQOL could not be determined. Further research is needed to investigate the causal relationships using experimental designs with workers or employees as the sample population. For example, Emerson et al. (2017) demonstrated that the effects of an exercise and nutrition workplace wellness program reduced stress and improved participants' quality of life. Furthermore, we did not analyze the occupation because we focused on understanding the overall relationship between exercise and physical activity and HRQOL among workers. However, since one study found a negative relationship between sitting time and self-rated health (Wilson et al., 2019), research on the relationship between occupation or job content and HRQOL may be needed in the future. Third, we asked the participants to answer the questions about exercise and physical activity time in a matrix format among the questions about lifestyle. After the question about exercise time, the respondents were asked to answer questions regarding physical activity (including work) to distinguish between exercise and other physical activities. However, since this was a self-administered questionnaire survey, some respondents may have different perceptions of physical activity (e.g., they may have limited their answers about physical activity time to time spent at work). Furthermore, the questionnaire only asked about the duration of exercise during leisure and physical activity, including work, not the intensity of exercise and physical activity. Fourth, this study was conducted during the COVID-19 pandemic. As such, the impact of exercise and physical activity on HRQOL needs to be considered within the environmental context of the pandemic, which might differ under normal circumstances. Considering that the duration of physical activity was affected by the COVID-19 pandemic and that the participants were anxious about the infection, a continuous evaluation is necessary.

## Conclusions

This study investigated the relationship between exercise time during leisure, physical activity time, and HRQOL. These results suggest that exercise time during leisure is associated with better HRQOL than physical activity time, including work. Interventions that encourage workers with no exercise habit to exercise daily, even for a short time, are likely to be associated with better workers' health and work performance.

## Data Availability Statement

The datasets presented in this article are not readily available because, the nature of this research, the participants of this study did not agree to their data being publicly shared, and hence, supporting data was not available. Requests to access the datasets should be directed to Ryosuke Sugano, ryosuke-sugano@med.uoeh-u.ac.jp.

## Ethics Statement

The studies involving human participants were reviewed and approved by The Ethics Committee of the University of Occupational and Environmental Health, Japan (No. R2-079). The patients/participants provided their written informed consent to participate in this study.

## Author Contributions

RS wrote the manuscript and analyzed the data. KI reviewed the manuscript, created the questionnaire, analyzed data, and provided advice on interpretation. HE, MT, ST, TN, and SM reviewed the manuscript. YF reviewed the manuscript and contributed to overall survey planning, creating the questionnaire, and securing funding for research. AO reviewed the manuscript, analyzed data, provided advice on interpretation, and secured funding for research. All authors contributed to the article and approved the submitted version.

## Funding

This study was funded by a research grant from the University of Occupational and Environmental Health, Japan; a general incorporated foundation (Anshin Zaidan) for the development of educational materials on mental health measures for managers at small-sized enterprises; Health, Labour, and Welfare Sciences Research Grants including Comprehensive Research for Women's Healthcare (H30-josei-ippan-002) and Research for the Establishment of an Occupational Health System during a disaster (H30-roudou-ippan-007); and scholarship donations from Chugai Pharmaceutical Co., Ltd.

## Conflict of Interest

The authors declare that the research was conducted in the absence of any commercial or financial relationships that could be construed as a potential conflict of interest.

## Publisher's Note

All claims expressed in this article are solely those of the authors and do not necessarily represent those of their affiliated organizations, or those of the publisher, the editors and the reviewers. Any product that may be evaluated in this article, or claim that may be made by its manufacturer, is not guaranteed or endorsed by the publisher.

## Abbreviations

HRQOL: health-related quality of life
WHO: World Health Organization
COVID-19: coronavirus disease 2019
CDC: centers for disease control and prevention
GLMs: generalized linear models.
